# Ginsenoside Rh4 Triggers Ferroptosis in Lung Cancer: Targeting *KEAP1*/*NRF2*/*HO-1* and Remodeling Gut Microbiota for Butyrate-Mediated *ATF3* Activation

**DOI:** 10.3390/ijms27062703

**Published:** 2026-03-16

**Authors:** Qihan Zhu, Wenxuan Xu, Ge Yang, Yansong Gao, Yujuan Zhao, Zijian Zhao, You Kang, Shengyu Li, Lei Zhao

**Affiliations:** 1 School of Pharmaceutical Sciences, Changchun University of Chinese Medicine, Changchun 130117, China; 2 Institute of Agro-food Technology, Jilin Academy of Agricultural Sciences (Northeast Agricultural Research Center of China), Changchun 130033, China

**Keywords:** ginsenoside Rh4, Lewis lung carcinoma, ferroptosis, oxidative damage, gut microbiota, butyrate

## Abstract

Lung cancer progression is regulated by multiple factors, including ferroptosis and gut microbiota-mediated butyrate metabolism. This study investigates the anti-tumor effects of ginsenoside Rh4 on lung cancer cells via ferroptosis mechanisms in vitro and in vivo. In vitro, ginsenoside Rh4 inhibited the proliferation of Lewis lung carcinoma (LLC) and A549 cells and triggered ferroptosis, effects that were suppressed by the ferroptosis inhibitor Ferrostatin-1 (Fer-1). In vivo, tumor-bearing mouse models were established and treated with 100 mg/kg ginsenoside Rh4 for 21 days. Tumor growth, ferroptosis markers, gut microbiota, and butyrate were analyzed, with in vitro validation of butyrate’s pathway effects. Ginsenoside Rh4 induced ferroptosis in LLC cells both in vitro and in vivo, inhibiting tumor growth. It promoted ferroptosis by disrupting iron homeostasis through elevated Fe^2+^ and transferrin receptor (*TFRC*), and impaired antioxidant defense via depletion of glutathione (*GSH*) and reduction in ferritin heavy chain 1 (*FTH1*), solute carrier family 40 member 1 (*SLC40A1*), solute carrier family 7 member 11 (*SLC7A11*), and glutathione peroxidase 4 (*GPX4*). Additionally, ginsenoside Rh4 enhanced lipid peroxidation, indicated by increased lipid peroxides (*LPO*) and malondialdehyde (*MDA*). In vivo, it suppressed the *KEAP1*/*NRF2*/*HO-1* pathway, reducing antioxidant enzyme activity. Gut microbiota modulation and butyrate production further amplified ferroptosis by activating transcription factor 3 (*ATF3*)-mediated *GPX4* suppression. Ginsenoside Rh4 induces ferroptosis by inhibiting the *KEAP1*/*NRF2*/*HO-1* pathway and remodeling the gut microbiota to increase butyrate levels, which synergistically enhance tumor cell ferroptosis sensitivity through *ATF3* activation and suppression of *GPX4*.

## 1. Introduction

Lung cancer has a high global incidence and malignancy, seriously endangering human health [1]. With approximately 85% of lung cancer cases being classified as such, non-small cell lung cancer (NSCLC) is the predominant histological subtype [2]. Major risk factors for NSCLC include smoking, genetic predisposition, and chronic lung infections [3]. However, traditional chemotherapy for NSCLC faces challenges like high drug toxicity and resistance. As a result, natural products—because of their lower toxicity and ability to target multiple pathways—are increasingly studied for preventing and treating NSCLC.

Ferroptosis involves iron-driven lipid peroxidation and oxygen species (ROS) accumulation, distinguishing it from apoptosis, necrosis, and autophagy, and highlighting its therapeutic potential in cancer [4]. NSCLC cells, characterized by high iron dependence and low ROS tolerance, are particularly vulnerable to ferroptosis [5]. The hallmark features of ferroptosis are the excessive buildup of lipid peroxides (*LPO*) and Fe^2+^ overload, regulated by antioxidant pathways and iron homeostasis [4]. Antioxidant defense mainly depends on glutathione peroxidase 4 (*GPX4*), whose activity requires glutathione (*GSH*) synthesized from cysteine imported via the cystine/glutamate antiporter (*SLC7A11*/xCT). Inhibiting the *SLC7A11*/*GSH*/*GPX4* pathway triggers ferroptosis. Regarding iron balance, transferrin receptor (*TFRC*) promotes iron uptake, which drives ferroptosis, while solute carrier family 40 member 1 (*SLC40A1*) counteracts this process by exporting iron [6]. Notably, the Kelch-like ECH-associated protein 1/nuclear factor erythroid 2-related factor 2 (*KEAP1*/*NRF2*) antioxidant axis is a key negative regulator of ferroptosis. Frequent loss-of-function mutations in *KEAP1* in NSCLC lead to constitutive activation of *NRF2*, which upregulates a suite of cytoprotective genes (including *SLC7A11* and *FTH1*), thereby conferring resistance to ferroptosis and posing a significant clinical challenge [7]. Therefore, targeting the *NRF2*/*GPX4* pathway to induce ferroptosis represents a new therapeutic approach. However, the efficacy of ferroptosis induction can be profoundly influenced by systemic factors, including the gut microbiota and its metabolites, which have recently emerged as master regulators of this cell death process.

Recent studies indicate that the gut microbiota significantly influences NSCLC development. Gut microbes affect NSCLC progression through mechanisms including host immune response modulation, inflammation suppression, and metabolic regulation [8]. Furthermore, the gut microbiota has been found to inhibit tumor growth by remodeling the microbial community to mediate immune cell infiltration into tumors and to regulate ferroptosis [9]. Conversely, specific pathogenic gut bacteria, such as *Fusobacterium nucleatum*, have been demonstrated to inhibit ferroptosis and confer chemotherapy resistance in colorectal cancer, highlighting the dual role of microbiota in regulating this cell death process [10]. Notably, during their metabolic processes, gut microorganisms produce various metabolites that interact with host cells. Emerging evidence indicates that gut microbiota-derived metabolites are involved in the occurrence of ferroptosis and participate in the progression of tumor diseases. For instance, microbial metabolites such as short-chain fatty acids (SCFAs) can enhance tumor cell susceptibility to ferroptosis [11]. Butyrate, a major SCFA, possesses anti-inflammatory, immunomodulatory, and anticancer activities. Research has shown that butyrate can inhibit tumor growth and induce cell death in multiple malignancies, such as lung cancer. Importantly, butyrate has been demonstrated to indirectly inhibit the expression of *SLC7A11*, which promotes ferroptosis in endometrial cancer [12]. These findings collectively establish gut microbiota-derived metabolites as key regulators of disease progression and therapeutic response by mediating ferroptosis. However, their function in lung cancer remains unclear. Currently, it remains poorly understood whether alterations in the gut microbiota and butyrate directly contribute to ferroptosis resistance in NSCLC cells and what their specific roles are in NSCLC progression.

Ginsenoside Rh4, a rare type of protopanaxatriol saponin found in the plant *Panax ginseng*, has anti-inflammatory, anticancer, and immunomodulatory activities [13]. The anti-tumor activity of ginsenoside Rh4 has been demonstrated in several malignancies, such as colon cancer, myeloma, and breast cancer. Research on lung cancer has demonstrated that ginsenoside Rh4 inhibits the JAK2/STAT3 axis, hence suppressing the spread of lung adenocarcinoma [14]. Notably, ferroptosis has been identified as a key mechanism underlying ginsenoside Rh4’s anti-tumor activity, involving pathways such as SIRT2 inhibition or p53 activation and autophagy [15,16]. Given its multi-target potential, we hypothesized that ginsenoside Rh4 might exert its anti-NSCLC effects not only by directly inducing tumor cell ferroptosis but also by potentially modulating the gut microbiota-butyrate axis to create a tumor microenvironment more permissive to ferroptosis. However, the specific mechanism by which ginsenoside Rh4 inhibits lung cancer through ferroptosis induction and whether it involves gut microbiota remodeling remains unclear. Thus, the current work assessed the effect of ginsenoside Rh4 on ferroptosis-related proteins and genes as well as cell survival in mouse Lewis lung cancer (LLC) and A549 cells in vitro. In parallel, an LLC tumor-bearing mouse model was created to evaluate Ginsenoside Rh4’s in vivo anti-tumor activity and its capacity to trigger ferroptosis via the *KEAP1*/*NRF2*/*HO-1* axis. Additionally, 16S rRNA analysis assessed alterations in the gut microbiota of tumor-bearing mice, and butyrate concentration among SCFAs was measured. This further explores how butyrate enhances tumor cell sensitivity to ginsenoside Rh4-induced ferroptosis by activating transcription factor 3 (*ATF3*) and suppressing *GPX4* expression.

## 2. Results

### 2.1. Ginsenoside Rh4 Promotes Ferroptosis-Associated Changes in LLC and A549 Cells

**Figure 1:**
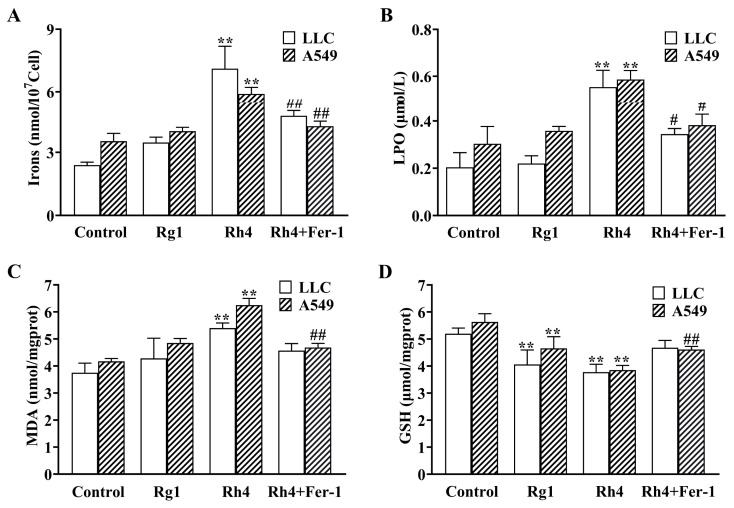
Effect of ginsenoside Rh4 on levels associated with ferroptosis in LLC and A549 cells Effects of Rg1, Rh4, and Rh4 combined with Fer-1 on intracellular Fe^2+^ (**A**), *LPO* (**B**), *MDA* (**C**), and *GSH* (**D**) levels in LLC and A549 cells. All data are expressed as mean ± standard deviation (SD) (*n* = 3). Compared with the Control group, ** *p* < 0.01; compared with Rh4 group: # *p* < 0.05, ## *p* < 0.01.

**Figure 2:**
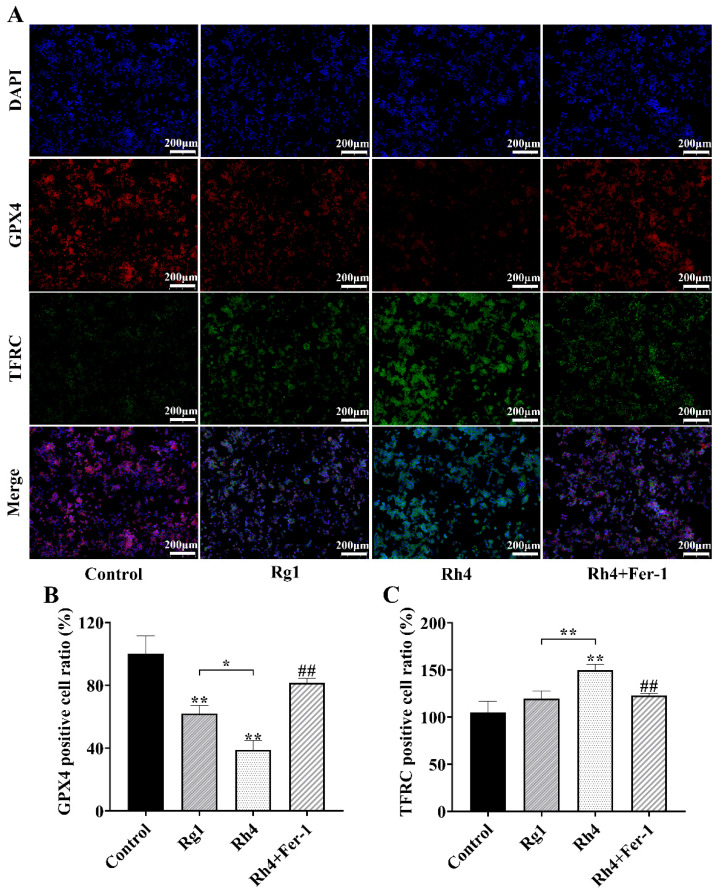
Ginsenoside Rh4 affects the proportion of *GPX4* and *TFRC*-positive cells (**A**) Representative immunofluorescence images. (**B**,**C**) Quantitative analysis of *GPX4* and *TFRC*-positive cells. All data are expressed as mean ± SD (*n* = 3). Compared with the Control group, * *p* < 0.05, ** *p* < 0.01; compared with Rh4 group: ## *p* < 0.01.

To evaluate the anti-tumor effects of ginsenosides Rg1 and Rh4, we first assessed their inhibitory activity on the proliferation of LLC and A549 cells. As shown in Figure S1A,B, treatment with ginsenoside Rh4 for 24 h inhibited the proliferation of both LLC and A549 cells in a dose-dependent manner within the tested concentration range. In comparison, ginsenoside Rh4 exhibited significantly stronger inhibitory effects compared to the unfermented ginsenoside Rg1. The IC_50_ values of Rh4 in LLC and A549 cells were 54.61 μg/mL and 55.75 μg/mL, respectively. To investigate the underlying mechanism, we examined markers of ferroptosis. As demonstrated in Figure 1A,B, compared with the control group, ginsenoside Rh4 increased the Fe^2+^ concentration in LLC and A549 cells from 2.39 ± 0.13 nmol/10^7^ cells and 3.53 ± 0.37 nmol/10^7^ cells to 7.10 ± 1.07 nmol/10^7^ cells and 5.90 ± 0.31 nmol/10^7^ cells, respectively. Similarly, the *LPO* level rose from 0.20 ± 0.06 μmol/L and 0.30 ± 0.07 μmol/L to 0.55 ± 0.07 μmol/L and 0.59 ± 0.04 μmol/L in the two cell lines. In contrast, the Rg1 group showed no significant differences compared with the control. The *MDA* content in the Rh4-treated group was significantly higher than that in the control, with increases of 44.00 ± 14.2% and 49.83 ± 4.10% in LLC and A549 cells, respectively (Figure 1C). Both Rg1 and Rh4 treatment resulted in significantly reduced glutathione levels compared with the control; however, the decrease was markedly greater in the Rh4 group than in the Rg1 group (Figure 1D). Notably, the ferroptosis inhibitor Ferrostatin-1 (Fer-1) largely reversed these Rh4-induced changes, confirming the involvement of ferroptosis in its anti-tumor activity. Furthermore, immunofluorescence analysis revealed that compared with the strong *GPX4* fluorescence observed in the control and Rg1 groups, ginsenoside Rh4 treatment significantly reduced *GPX4* signal intensity. Conversely, the *TFRC* signal was markedly enhanced in the Rh4 group (Figure 2A–C). Quantitative analysis showed that after treatment with ginsenoside Rh4, the proportion of *GPX4*-positive cells decreased to 38.93 ± 5.8%, while the proportion of *TFRC*-positive cells increased by 40.29 ± 2.0% relative to the control group. Importantly, these Rh4-induced changes were substantially attenuated by co-treatment with the ferroptosis inhibitor Fer-1, confirming the specific role of ferroptosis in this process.

### 2.2. Ginsenoside Rh4 Affects the Expression of Ferroptosis-Related Proteins and Genes in LLC Cells

**Figure 3:**
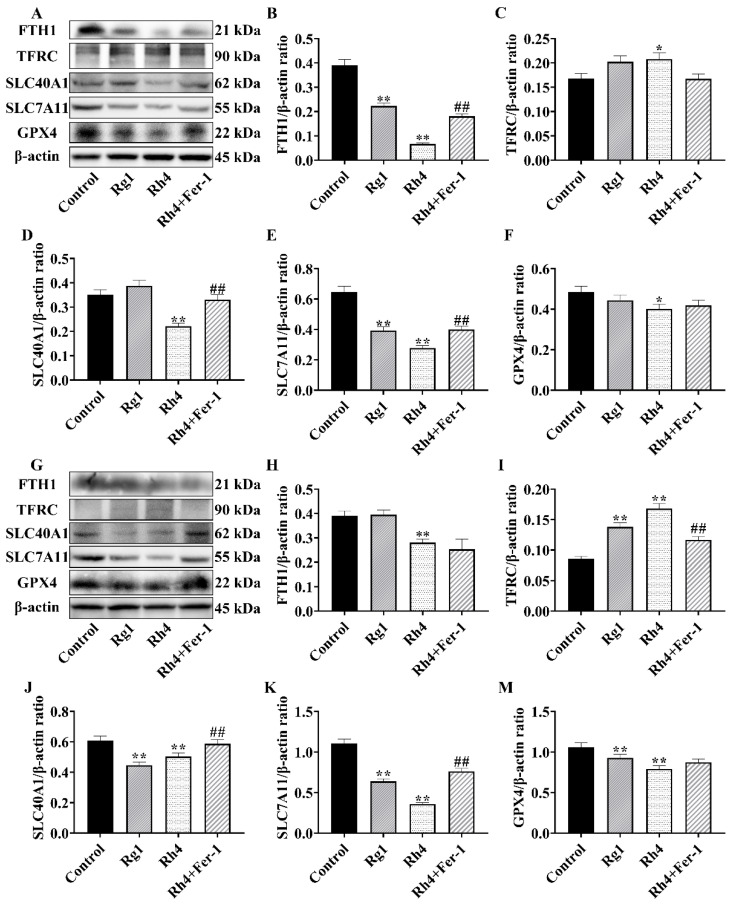
Ginsenoside Rh4 affects the expression of ferroptosis-related proteins and genes in LLC and A549 cells (**A**) Representative Western blot images of LLC cells. (**B**–**F**) Relative protein expression levels of *FTH1*, *TFRC*, *SLC40A1*, *SLC7A11*, *GPX4*, and β-actin proteins in LLC cells. (**G**) Representative Western blot images of A549 cells. (**H**–**M**) Relative protein expression levels of *FTH1*, *TFRC*, *SLC40A1*, *SLC7A11*, *GPX4*, and β-actin proteins in A549 cells. All data are expressed as mean ± SD (*n* = 3). Compared with the Control group, * *p* < 0.05, ** *p* < 0.01; compared with Rh4 group: ## *p* < 0.01.

**Figure 4:**
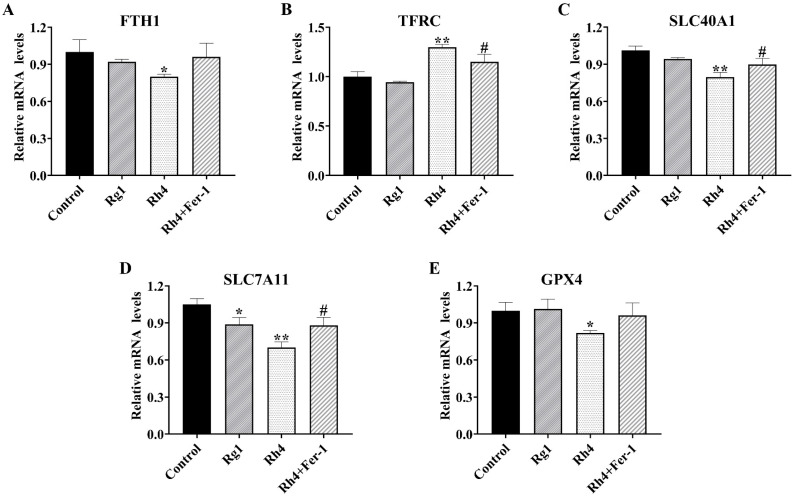
RT-qPCR detection of relative mRNA expression of *FTH1* (**A**), *TFRC* (**B**), *SLC40A1* (**C**), *SLC7A11* (**D**), and *GPX4* (**E**) in LLC cells. All data are expressed as mean ± SD (*n* = 3). Compared with the Control group, * *p* < 0.05, ** *p* < 0.01; compared with Rh4 group: # *p* < 0.05.

To further elucidate the molecular mechanism underlying ginsenoside Rh4-induced ferroptosis, we analyzed the expression of key ferroptosis-regulatory proteins in LLC (Figure 3A–F) and A549 (Figure 3G–M) cells by Western blotting. In LLC cells, ginsenoside Rh4 treatment significantly upregulated *TFRC* while downregulating *FTH1*, *SLC40A1*, *SLC7A11*, and *GPX4*. These changes were substantially reversed by co-treatment with the ferroptosis inhibitor Fer-1. A consistent expression profile was observed in A549 cells. Ginsenoside Rh4 similarly increased *TFRC* and decreased *FTH1*, *SLC40A1*, *SLC7A11*, and *GPX4*, effects that were again attenuated by Fer-1. We next investigated whether Rh4 regulates ferroptosis at the transcriptional level. qRT-PCR analysis of key ferroptosis-related genes in LLC cells revealed that Rh4 significantly downregulated mRNA expression of *FTH1* (*p* < 0.05), *SLC40A1* (*p* < 0.01), *SLC7A11* (*p* < 0.01), and *GPX4* (*p* < 0.05), while upregulating *TFRC* (*p* < 0.01) compared with the control (Figure 4A–E). These transcriptional changes were largely reversed upon co-treatment with Fer-1.

### 2.3. Ginsenoside Rh4 Inhibits Tumor Growth in LLC Tumor-Bearing Mice

**Figure 5:**
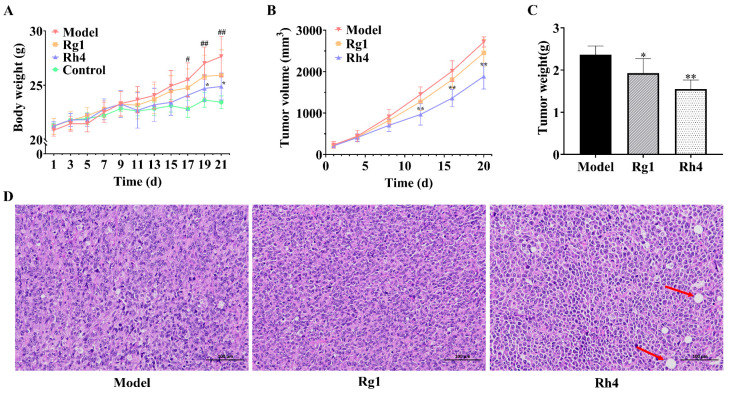
Inhibitory effect of ginsenoside Rh4 on tumor growth in LLC tumor-bearing mice Effects of ginsenoside Rh4 on body weight (**A**), tumor volume (**B**), and tumor weight (**C**) in LLC tumor-bearing mice. (**D**) H&E staining of mouse tumor tissue. All data are expressed as mean ± SD (*n* = 12). Compared with the Control group, # *p* < 0.05, ## *p* < 0.01. Compared with the Model group, * *p* < 0.05, ** *p* < 0.01.

As shown in Figure 5A, the body weight of mice in the Rh4 group was significantly lower than that in the model group (*p* < 0.05). Concurrently, Figure 5B demonstrates that treatment with ginsenoside Rg1 and ginsenoside Rh4 reduced tumor volume compared to the model group, with the Rh4 group exhibiting the greatest reduction of 30.65% (*p* < 0.01). Consistent with the tumor volume changes, the Rh4 group also showed the lowest tumor weight (Figure 5C) and achieved a tumor inhibition rate of 34.32% (*p* < 0.01) relative to the model group. These results indicate that ginsenoside Rh4 demonstrates superior efficacy compared to ginsenoside Rg1. Furthermore, compared to the model group, H&E staining results (Figure 5D) demonstrated disrupted tumor architecture, visible necrotic areas, loosely arranged cells, and evident pyknosis in the Rh4 group. Therefore, ginsenoside Rh4 effectively inhibited the growth of LLC cells in vivo.

### 2.4. Ginsenoside Rh4 Modulates Changes in Iron Homeostasis and Ferroptosis in LLC Tumor-Bearing Mice

**Figure 6:**
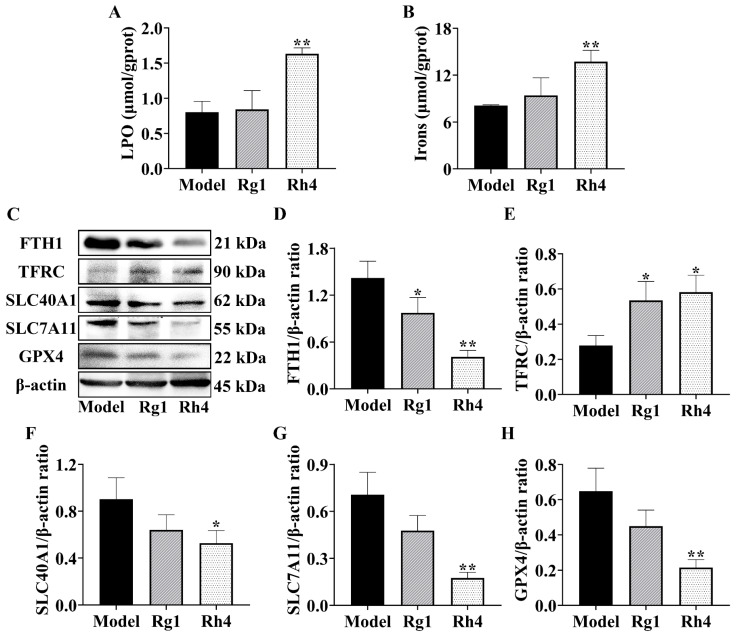
Effects of ginsenoside Rh4 on ferroptosis-related pathways in LLC-bearing mice (**A**) *LPO* levels in tumor tissues (*n* = 12). (**B**) Iron concentration in tumor tissues (*n* = 12). (**C**) Representative Western blot images. (**D**–**H**) Relative protein expression levels of *FTH1*, *TFRC*, *SLC40A1*, *SLC7A11*, *GPX4*, and β-actin proteins in mouse tumors (*n* = 3). All data are expressed as mean ± SD. Compared with the Model group, * *p* < 0.05, ** *p* < 0.01.

Subsequently, levels of *LPO*, iron concentration, and ferroptosis-related proteins were measured in mouse tumor tissues. As shown in Figure 6A,B, compared to the model group, the Rh4 group exhibited increased *LPO* levels (1.63 ± 0.08 vs. 0.80 ± 0.15 μmol/gprot) and elevated iron concentration (13.73 ± 1.45 vs. 8.10 ± 0.64 μmol/gprot) in tumor tissues. Furthermore, Western blotting (Figure 6C–H) revealed that compared to the model group, ginsenoside Rh4 treatment significantly reduced the levels of *FTH1*, *SLC40A1*, *SLC7A11*, and *GPX4* by 40.96%, 52.71%, 17.55%, and 21.53%, respectively, and increased *TFRC* levels by 58.27%. Compared with ginsenoside Rg1, the effects of ginsenoside Rh4 were more pronounced. These findings are consistent with the in vitro results.

### 2.5. Ginsenoside Rh4 Downregulates KEAP1/NRF2/HO-1 Axis in LLC Tumor-Bearing Mice

**Figure 7:**
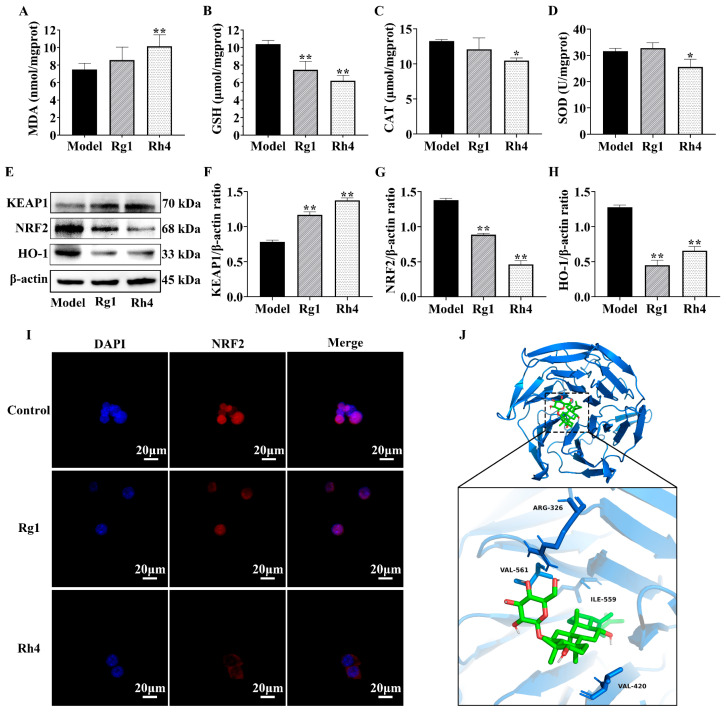
Effects of ginsenoside Rh4 on tumor oxidative damage and *KEAP1*/*NRF2*/*HO-1* pathway expression in LLC tumor-bearing mice (**A**–**D**) *MDA*, *GSH*, CAT, and SOD levels in tumor tissues (*n* = 12). (**E**) Representative Western blot images. (**F**–**H**) Relative protein expression levels of *KEAP1*, *NRF2*, *HO-1*, and β-actin proteins in mouse tumors (*n* = 3). (**I**) Immunofluorescence staining for detecting *NRF2* levels in LLC cells (*n* = 3). (**J**) Three-dimensional binding mode analysis of ginsenoside Rh4 and *KEAP1*. All data are expressed as mean ± SD. Compared with the Model group, * *p* < 0.05, ** *p* < 0.01.

The *NRF2* antioxidant pathway is aberrantly activated in LLC cells and promotes the malignant progression of lung cancer through multiple mechanisms. Since ferroptosis can be suppressed by the *NRF2*/*HO-1* pathway, we investigated whether ginsenoside Rh4 could inhibit the *NRF2* pathway to induce ferroptosis. As shown in Figure 7A–D, compared to the model group, the Rh4 group exhibited significantly increased *MDA* content and decreased considerably activities of *GSH*, CAT, and SOD in tumor tissues. Concurrently, Western blot analysis (Figure 7E–H) revealed that Rh4 intervention significantly increased *KEAP1* protein levels and significantly decreased *NRF2* and heme oxygenase-1 (*HO-1*) protein levels compared to the model group. In tumor cells, *NRF2* is typically localized and highly expressed in the nucleus. Immunofluorescence analysis of LLC cells demonstrated that ginsenoside Rh4 significantly reduced overall *NRF2* expression and inhibited its nuclear translocation (Figure 7I). Molecular docking studies further indicated that ginsenoside Rh4 binds directly to *KEAP1* with a docking score of −9.755 kcal/mol. PyMOL (version 3.1) visualization revealed that Rh4 forms hydrogen bonds with key residues of *KEAP1*, including ARG-326, VAL-561, ILE-559, and VAL-420 (Figure 7J).

### 2.6. Ginsenoside Rh4 Reverses Gut Microbiota Dysbiosis in LLC Tumor-Bearing Mice

**Figure 8:**
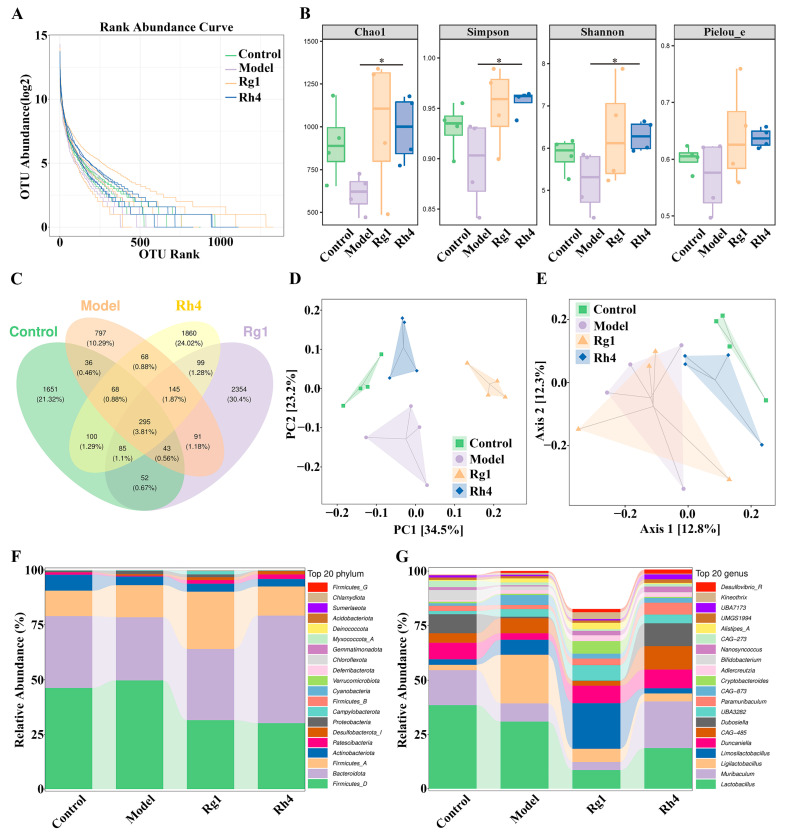
Ginsenoside Rh4-mediated changes in the gut microbiota of LLC tumor-bearing mice. (**A**) OTU abundance rank curves. (**B**) Chao1, Simpson, Shannon, and Pielou_e indices. (**C**) Venn plot of OTUs. (**D**,**E**) PCA and PCoA results of different bacterial taxa at the OTU level. (**F**) Relative abundance of gut microbiota at the phylum level. (**G**) Relative abundance of gut microbiota at the genus level. All data are expressed as mean ± SD (*n* = 4). Compared with the Model group, * *p* < 0.05.

The effect of ginsenoside Rh4 on gut microbiota was evaluated through 16S rRNA sequencing, with sample abundance rank curves presented in Figure 8A. The gentle slope and broad horizontal range of the curves indicate high evenness and low variation in abundance among species within the samples. Alpha diversity analysis (Figure 8B) revealed that, compared to the model group, the Rh4 group exhibited significantly increased Chao 1 (*p* < 0.05), Simpson (*p* < 0.05), and Shannon (*p* < 0.05) indices. Venn analysis identified a total of 7744 OTUs, with 548 OTUs shared between the Rh4 and control groups, indicating high similarity between these groups (Figure 8C). Beta diversity analysis, represented by PCA and PCoA, demonstrated that the microbial community structure in the Rh4 group shifted closer to that of the control group after ginsenoside Rh4 treatment (Figure 8D,E). Following treatment with ginsenoside Rh4, there was an increase in *Bacteroidota* and a decrease in *Firmicutes* and *Proteobacteria* at the phylum level (Figure 8F). At the genus level, intervention with ginsenoside Rh4 increased the relative abundances of beneficial bacteria, such as *Muribaculum*, *Duncaniella*, *CAG-485*, *Dubosiella*, *Paramuribaculum*, and *UBA3282*, while decreasing the relative abundances of deleterious bacteria, including *Lactobacillus*, *Ligilactobacillus*, and *Limosilactobacillus*, bringing them closer to the levels observed in the control group (Figure 8G).

### 2.7. Ginsenoside Rh4 Reshapes Gut Microbiota Functionality and Intestinal SCFAs

**Figure 9:**
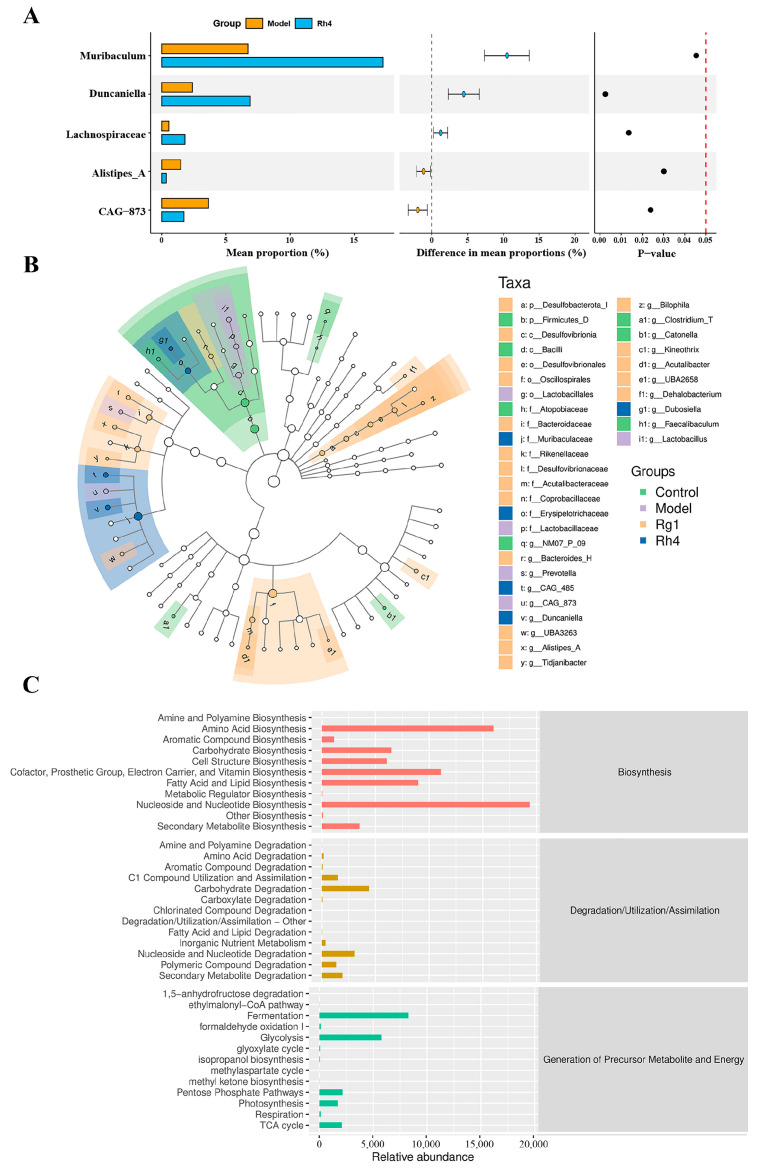
Ginsenoside Rh4 induces between-group differences in gut microbiota abundance. (**A**) Differential abundance of key bacterial taxa: left bars show mean proportion (Model: orange, Rh4: blue), middle points/error bars show the difference in mean proportions (colored consistently with the corresponding group), and right dots show P-values (dashed line at *p* = 0.05). (**B**) Branching figure of LefSe. (**C**) Relative abundance of microbial functional pathways, with colors denoting functional categories: red (Biosynthesis), gold (Degradation/Utilization/Assimilation), and teal (Generation of Precursor Metabolite and Energy). All data are expressed as mean ± SD (*n* = 4).

To identify key bacterial species involved in ginsenoside Rh4-mediated lung cancer suppression, we investigated the effect of ginsenoside Rh4 administration on the gut microbiota in mice. As shown in Figure 9A, compared to the model group, ginsenoside Rh4 treatment significantly increased the relative abundances of *Muribaculaceae*, *Dubosiella*, and *Lachnospiraceae* (*p* < 0.05). This finding was corroborated by Linear Discriminant Analysis Effect Size (LEfSe) analysis (Figure 9B). Furthermore, functional profiling based on KEGG pathway abundance indicated that the primary pathways modulated by ginsenoside Rh4 were associated with fatty acid and lipid biosynthesis, fermentation, and glycolysis (Figure 9C). Subsequently, the levels of SCFAs in mouse colonic contents were quantified. As shown in Table 1, compared with the model group, the Rh4 group exhibited significantly elevated levels of SCFAs, with butyric acid levels increasing dramatically by 70.50% following ginsenoside Rh4 administration (*p* < 0.05).

### 2.8. Gut Microbiota, SCFAs, and Cell Death Factors from Multifactorial Correlation Analysis

**Figure 10:**
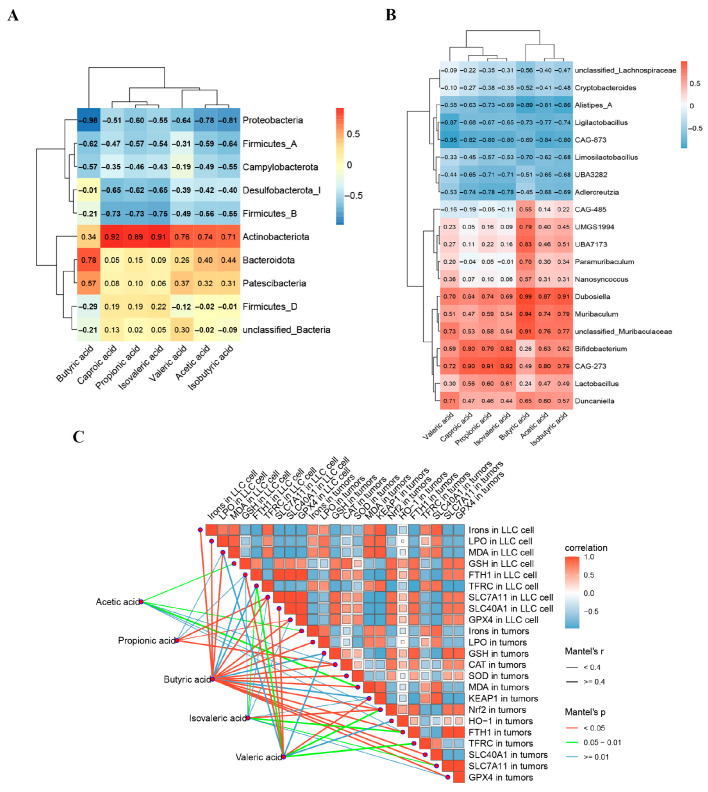
Combined analysis of gut microbiota, ferroptosis, and SCFAs and multifactorial correlation analysis. (**A**) Correlation between gut microbiota at the phylum level and SCFAs in mice. (**B**) Correlation between gut microbiota at the genus level and butyric acid in mice. (**C**) Correlation analysis among oxidative stress markers, ferroptosis-related factors, and SCFAs in mice.

Correlation analyses were performed between gut microbiota and SCFAs. As shown in Figure 10A, at the phylum level, *Proteobacteria* and *Firmicutes* were negatively correlated with SCFAs, while *Actinobacteriota* was positively correlated. Specifically, *Proteobacteria* was negatively correlated with butyric acid, and *Bacteroidota* was positively correlated. At the genus level (Figure 10B), *Alistipes_A* was negatively correlated with butyric acid, whereas *UBA7173*, *Dubosiella*, and *Muribaculum* showed significant positive correlations. Subsequently, correlations between oxidative factors, ferroptosis-related indicators, and SCFAs were examined. Heatmap analysis (Figure 10C) revealed that the oxidative damage markers *MDA* and Keap1 were negatively correlated with the anti-ferroptosis factors *FTH1*, *SLC7A11*, *SLC40A1*, and *GPX4*. Moreover, butyric acid among the SCFAs exhibited significant correlations with ferroptosis-related factors, including iron, *LPO*, *SLC7A11*, and *GPX4* in both LLC cells and mice.

### 2.9. Butyrate and Ginsenoside Rh4 Activate Ferroptosis in LLC Cells in Vitro via the ATF3/SLC7A11/GPX4 Pathway

**Figure 11:**
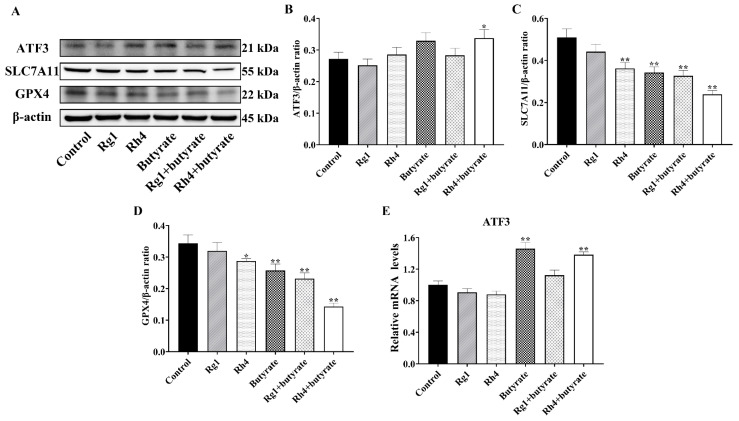
Butyrate enhances the sensitivity to ferroptosis in LLC cells. (**A**–**D**) Protein levels of *ATF3*, *SLC7A11*, *GPX4*, and β-actin were determined using Western blot in LLC cells. (**E**) RT-qPCR detection of relative mRNA expression of *ATF3* in LLC cells. All data are expressed as mean ± SD (*n* = 3). Compared with the Control group, * *p* < 0.05, ** *p* < 0.01.

The effect of butyrate on ferroptosis in LLC cells was subsequently investigated in vitro. CCK-8 assays revealed that butyrate exerted cytotoxic effects on LLC cells at approximately 1.5 μm (Figure S2A). Furthermore, co-treatment with butyrate and ginsenoside Rh4 significantly enhanced the inhibition rate of cell viability (Figure S2B). Furthermore, as shown in Figure 11A–E, Western blot and RT-qPCR analyses revealed that butyrate treatment alone increased both mRNA and protein levels of *ATF3*, while it decreased protein expression of *SLC7A11* and *GPX4* compared with the control group. In comparison to the control, combined treatment with butyrate and ginsenoside Rh4 resulted in a 6.61% increase in *ATF3* protein expression, a 27.10% decrease in *SLC7A11* protein expression, and a 20.03% reduction in *GPX4* protein expression.

## 3. Discussion

Ferroptosis, a newly recognized form of programmed cell death, has attracted considerable attention in cancer research. Ginsenosides can influence tumor cells by modulating ferroptosis. The *SLC7A11*/*GSH*/*GPX4* signaling pathway is a key regulator of ferroptosis. *SLC7A11* imports extracellular cystine into cells for *GSH* synthesis, and *GPX4* utilizes *GSH* to reduce lipid peroxides, thereby inhibiting ferroptosis [17]. In this study, ginsenoside Rh4 was found to downregulate *SLC7A11* and *GPX4* protein expression, reduce cellular *GSH* levels, promote the accumulation of *LPO* and *MDA*, and consequently induce ferroptosis in lung cancer cells. Critically, the ferroptosis inhibitor Fer-1 significantly rescued Rh4-induced cell death, confirming the specific role of ferroptosis in this process. Previous studies indicate that ginsenoside CK also induces ferroptosis and inhibits tumor growth in liver cancer cells by suppressing the *SLC7A11*/*GPX4* pathway, which aligns with our findings [18]. However, not all ginsenosides act via identical mechanisms. For instance, ginsenoside Rh2 was reported to indirectly suppress *SLC7A11* by upregulating IRF1 expression to induce ferroptosis, while ginsenoside F2 directly reduces cellular *GSH* levels and impairs *GPX4* function without significantly affecting *SLC7A11* [19,20]. Notably, excessive intracellular Fe^2+^ accumulation is a key trigger for ferroptosis, making the regulation of iron metabolism-related proteins crucial in ferroptosis-related diseases [21]. Furthermore, this study revealed that ginsenoside Rh4 upregulates the iron uptake receptor *TFRC*, while downregulating the iron storage protein *FTH1* and the iron export transporter *SLC40A1*, thereby promoting intracellular free iron accumulation and further activating ferroptosis in lung cancer cells. Additionally, ginsenoside Rg5 can induce ferroptosis by inhibiting HSPB1 and upregulating NCOA4 expression, which promotes *FTH1* degradation via the ferritinophagy pathway, increasing intracellular free iron levels and oxidative stress [22].

This study demonstrated that ginsenoside Rh4 treatment significantly reduced the activity of antioxidant enzymes, including SOD and CAT, and effectively suppressed lung cancer cell growth by targeting the *KEAP1*/*NRF2* signaling pathway. Specifically, ginsenoside Rh4 stabilized *KEAP1* protein levels by inhibiting its ubiquitin–proteasome pathway degradation. The enhanced *KEAP1*/*NRF2* interaction effectively prevented *NRF2* from escaping degradation and subsequent nuclear translocation. This mechanism aligns with reported findings where ginsenoside Rd inhibited both protein and mRNA expression of *NRF2* and its target genes *(NQO1*, *HO-1*, *GCLC*), exerting anticancer effects in lung cancer cells [23]. Notably, the effect of ginsenoside Rh4 on the *NRF2* pathway may differ across cell types and involve multiple mechanisms. In tumor cells, ginsenoside Rh4 stabilizes *KEAP1*, thereby downregulating *NRF2* activity to inhibit growth [16]. Conversely, in normal cells, it promotes *NRF2* nuclear translocation via Akt signaling activation, exerting cytoprotective effects [24]. Furthermore, the *NRF2* signaling pathway is a key negative regulator of ferroptosis. Its activation transcriptionally upregulates multiple critical anti-ferroptosis proteins, including *HO-1*, *SLC7A11*, *FTH1*, and *SLC40A1* [25]. This study further revealed that ginsenoside Rh4 treatment significantly decreased *HO-1* protein levels and increased intracellular iron content in lung cancer cells, thereby promoting ferroptosis. *HO-1* downregulation promotes ferroptosis through a dual mechanism: it weakens cellular antioxidant capacity, reducing ROS scavenging efficiency, and decreases the chelation of redox-active iron, leading to free iron accumulation and ferroptosis activation [26]. Studies have shown that ginsenoside Rh3 can mediate ferroptosis in colorectal cancer cells by activating the Stat3/p53/*NRF2*/*HO-1* signaling pathway [27].

**Figure 12:**
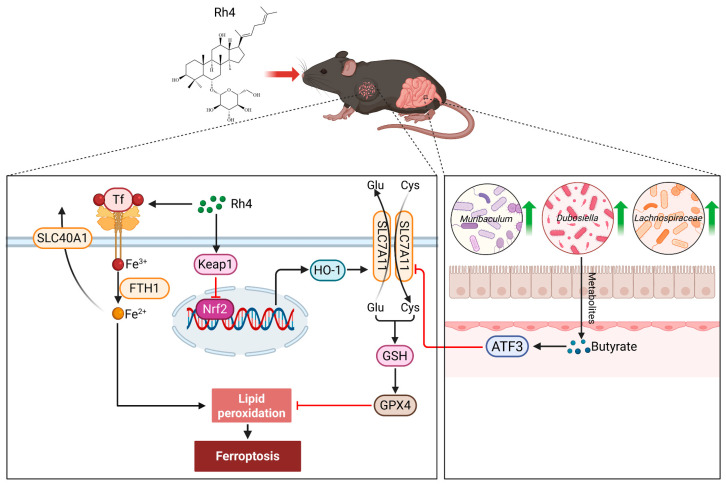
Ginsenoside Rh4 targets the *KEAP1*/*NRF2*/*HO-1* axis and restores gut microbiota to increase butyrate-mediated induction of ferroptosis via the *ATF3*/*SLC7A11*/*GPX4* pathway in lung cancer. Black solid arrows indicate activation or molecular transport, red T-shaped arrows indicate inhibitory effects.

Recent studies suggest that ginsenosides can modulate gut microbiota composition and abundance, and gut microbiota dysbiosis is associated with an increased risk of lung cancer and other diseases [28,29]. At the phylum level, the *Firmicutes*/*Bacteroidota* (F/B) ratio is closely associated with lung cancer development [30]. Our results show that ginsenoside Rh4 significantly reduced this ratio and decreased the abundance of the potentially harmful phylum *Proteobacteria*. Consistent with these findings, Bai et al. reported that ginsenoside Rk3 inhibited tumor growth by restoring microbiota homeostasis through reducing the F/B ratio [31]. At the genus level, ginsenoside Rh4 significantly increased the abundance of beneficial bacteria *Duncaniella* and *Paramuribaculum*, which are known for their anti-tumor activities [32]. In addition, this study observed that ginsenoside Rh4 treatment significantly reduced *Lactobacillus* abundance, weakened the AhR/Nrf2/*GPX4* signaling pathway, and promoted the activation of ferroptosis to inhibit tumor growth [33]. In contrast, ginsenoside CK treatment led to increased *Lactobacillus* abundance, regulated the intestinal barrier, and enhanced immunity [34]. This study further revealed that ginsenoside Rh4 significantly increased the abundances of *Muribaculum*, *Dubosiella*, and *Lachnospiraceae*, which are known to ferment complex carbohydrates into SCFAs, resulting in elevated overall SCFA levels. Notably, fecal butyrate levels were significantly elevated in ginsenoside Rh4-treated mice. Butyrate, a key SCFA, exhibits anti-inflammatory, immunomodulatory, and anti-tumor effects. Correlation analysis further revealed that increased butyrate levels were positively correlated with higher abundances of *Muribaculum* and *Lachnospiraceae*, consistent with previous reports identifying these microbes as major butyrate producers in the gut [35,36]. Additionally, earlier reports have shown that ginsenoside Rk3 can promote butyrate synthesis by enriching specific bacterial genera such as *Bacteroides* and *Alloprevotella*, thereby contributing to its anti-tumor effects [37]. These findings reveal gut microbiota alterations induced by ginsenoside Rh4, with potential mechanisms involving microbial metabolites or host immune responses warranting further investigation. Our in vitro experiments demonstrated that exogenous butyrate significantly enhanced ginsenoside Rh4-induced ferroptosis in LLC cells. Butyrate promoted this process by activating the *ATF3*/*SLC7A11*/*GPX4* signaling pathway (Figure 12). Additionally, other studies report that butyrate can inhibit the *SLC7A11*/*GPX4* pathway via upstream proteins (e.g., c-Fos) or directly promote *GPX4* ubiquitination and degradation to induce cancer cell ferroptosis [38,39].

## 4. Materials and Methods

### 4.1. Ginsenoside Rh4 Preparation

Ginsenoside Rh4 was prepared by biotransformation of ginsenoside Rg1 using *Lactiplantibacillus plantarum TRG22*, following the method reported by Shen et al. [40]. A total of 1 g of ginsenoside Rg1 (purchased from Shanghai Yuanye Biotechnology Co., Ltd., Shanghai, China) was dissolved in 1 L of sterile fermentation medium containing 30 g of glucose and 10 g of soybean oligopeptide. After filtering the solution using a 0.22 μm microporous filter, *L. plantarum TRG22* was added to the sterilized medium and allowed to ferment for 21 days at 37 °C. Centrifugation was used to gather the precipitate following fermentation, and it was then loaded onto a D101 macroporous resin column for purification. The column was eluted with gradient ethanol (0%, 30%, 55%, and 70% *v*/*v*), and the fractions eluted with 70% ethanol were concentrated under vacuum and subsequently freeze-dried. High-performance liquid chromatography (HPLC) revealed a single peak at 73.54 min, matching the retention time of reference standards for ginsenoside Rh4 (Shanghai Yuanye Biotechnology Co., Ltd., Shanghai, China) (Figure S3). The purity of the isolated product was determined to be 95.07% by an external standard method using a ginsenoside Rh4 reference standard calibration curve (y = 4948.8x − 6, R^2^ = 0.999). The isolated product was further confirmed to be ginsenoside Rh4 through liquid chromatography–mass spectrometry (LC-MS) as well as ^1^H and ^13^C nuclear magnetic resonance (NMR) spectroscopy (Figure S4). All spectroscopic data were in full agreement with published values for authentic ginsenoside Rh4 [41].

### 4.2. Cell Culture and Grouping

The LLC cells (Jiangsu Keygen BioTECH, Nanjing, China) and A549 cells (Jiangsu Keygen BioTECH, Nanjing, China) were kept in DMEM or RPMI 1640, respectively, supplemented with 10% fetal bovine serum and 1% penicillin–streptomycin. Cells were maintained in a humidified incubator at 37 °C with 5% CO_2_. Tour groups of LLC and A549 cells were created by seeding them in 6-well plates at a density of 1 × 10^5^ cells each: the Control group, the Rg1 group, the Rh4 group, and the Rh4 + Fer-1 group. A total of 100 μg/mL of ginsenoside Rg1 and ginsenoside Rh4 were incubated with the Rg1 and Rh4 groups for 24 h, while the control group was cultured with complete media. For the Rh4 + Fer-1 group, cells were pretreated with the ferroptosis inhibitor Ferrostatin-1 (Fer-1, 3 μM) for 6 h prior to the addition of 100 μg/mL ginsenoside Rh4, followed by co-incubation for 24 h as a rescue experiment.

### 4.3. Immunofluorescence Staining

LLC cells were harvested from the culture medium, fixed for 20 min in 4% paraformaldehyde, permeabilized for 15 min in 0.5% Triton X-100, and blocked for one hour in 3% bovine serum albumin. The cells were then incubated overnight with primary antibodies *GPX4* (Servicebio, Wuhan, China), *TFRC* (Proteintech, Wuhan, China) and *NRF2* (Proteintech, Wuhan, China) at 4 °C. The following day, after three rinses with phosphate-buffered saline (PBS), the cells were incubated with secondary antibodies Cy3-conjugated goat anti-rabbit IgG (Servicebio, Wuhan, China) and FITC-conjugated goat anti-rabbit IgG (Servicebio, Wuhan, China) for 1 h. The cells then underwent DAPI nuclear staining. Three repetitions of each experiment were made.

### 4.4. Quantitative Real-Time PCR (RT-qPCR)

Total RNA was extracted from LLC cells with Trizol reagent (Invitrogen, Carlsbad, CA, USA), followed by cDNA synthesis with a reverse transcription kit (Servicebio, Wuhan, China). The cDNA was then subjected to PCR amplification using specific primers, and the PCR products were detected with SYBR Green fluorescent dye in a CFX96 real-time quantitative PCR system (Servicebio, Wuhan, China). *GAPDH* was used as the reference gene, and analyzed by the 2^−ΔΔCT^ method. The primers used for the analysis were as follows: *FTH1* (forward: 5′-GATGTGGCTCTGAAGAACTTTGC-3′, reverse: 5′-CAGTCATCACGGTCTGGTTTCT-3′), *SLC40A1*(forward: 5′-GAGACAAGTCCTGAATCTGTGCC-3′, reverse: 5′-TTCTTGCAGCAACTGTGTCACAG-3′), *TFRC*(forward: 5′-TTAGTGATTGTTAGAGCAGGGGA-3′, reverse: 5′-GGCGGAAACTGAGTATGATTGA-3′), *SLC7A11*(forward: 5′-GCTATCATCACAGTGGGCTACG-3′, reverse: 5′-TAGAATAACCTGGAGACAGCGAAC-3′), *GPX4*(forward: 5′-GAGGCAGGAGCCAGGAAGTAA-3′, reverse: 5′-CACCACGCAGCCGTTCTTAT-3′).

### 4.5. Animals and Experimental Design

A total of 48 male C57BL/6 mice (20–22 g) were acclimated for 7 d in SPF-grade facilities. Experimental protocols were approved by the Institutional Animal Care Committee of Changchun University of Chinese Medicine (Ethics reference number: 2024881). The LLC tumor-bearing mouse model was established using the concentration reported by Zheng et al. through a subcutaneous injection of 1 × 10^6^ viable LLC cells suspended in 0.2 mL sterile PBS into the right axillary region [42]. When tumors reached approximately 500 mm^3^ (calculated as V = 0.5 × length × width^2^), mice were randomly allocated into four groups (*n* = 12/group): Control, Model, Rg1, and Rh4. Randomization was performed using a computer-generated random number sequence to ensure comparable baseline tumor volumes across groups. The Rg1 and Rh4 groups were gavaged with 100 mg/kg ginsenoside Rg1 and ginsenoside Rh4, while the control group and model group were gavaged with saline, respectively. Mice were treated for 21 d with daily monitoring of health and food intake. Body weight and tumor size were recorded every 2 d by investigators blinded to group allocation to minimize measurement bias. After the last administration, the mice were executed to collect the tumors for weighing, which was used to calculate the tumor inhibition rate (TIR), and spleen, lung, thymus, and fecal samples were harvested and stored at −80 °C. Sample processing and subsequent biochemical and molecular analyses were also performed in a blinded manner where feasible.

### 4.6. Biochemical Index Assay

LLC and A549 cells, as well as mouse tumor tissues, were lysed using RIPA lysis solution that contained protease inhibitors. A bicinchoninic acid (BCA) assay kit (Beyotime, Shanghai, China) was used to measure the protein content in the resultant supernatants. Using spectrophotometry, the amounts of Fe^2+^ and *LPO* in cells and tumor tissues were identified (Nanjing Jiancheng Bioengineering Institute, Nanjing, China). Using ELISA (Sangon Biotech, Shanghai, China), the amounts of malondialdehyde (*MDA*), *GSH*, catalase (CAT), and superoxide dismutase (SOD) in cells and tumor tissues were measured. 

### 4.7. Western Blotting

LLC and A549 cells, as well as mouse tumor tissues, were lysed with RIPA lysis solution containing protease inhibitors (Solarbio, Beijing, China) and the supernatant was collected. The protein content of the supernatant was measured and modified using the BCA kit (Beyotime, Shanghai, China). Protein samples were resolved on 10% SDS–polyacrylamide gels (UEIandy, Suzhou, China), transferred to PVDF membrane (Keygen BioTECH, Nanjing, China), and blocked with 5% bovine serum albumin. Primary antibodies against *FTH1* (Sangon Biotech, Shanghai, China), *GPX4* (Sangon Biotech, Shanghai, China), *HO-1* (Proteintech, Wuhan, China), *KEAP1* (Proteintech, Wuhan, China), *NRF2* (Proteintech, Wuhan, China), *SLC40A1* (Proteintech, Wuhan, China), *SLC7A11* (Sangon Biotech, Shanghai, China), *TFRC* (Proteintech, Wuhan, China), and β-actin (Proteintech, Wuhan, China) were incubated with the membrane at 4 °C overnight. Following an hour of room temperature incubation with an HRP-conjugated goat anti-rabbit secondary antibody (Proteintech, Wuhan, China), protein bands were identified using a ChemiScope 5300 (Clinx Science Instruments Co., Ltd, Shanghai, China). Signal intensity was quantified using ImageJ software (version 1.53).

### 4.8. Hematoxylin and Eosin (H&E) Staining

Tumor tissues underwent 24 h fixation in 4% paraformaldehyde at ambient temperature, followed by paraffin embedding and microtome sectioning. Sections were deparaffinized with xylene, soaked in an ethanol gradient, and washed with ultrapure water. They were stained with H&E, sealed with neutral adhesive, and visualized with a light microscope.

### 4.9. Gut Microbiota Analysis

To extract total DNA from the contents of the mouse colon, the QIAamp Fast Stool DNA Mini Kit (QIAGEN, Hilden, Germany) was utilized. Using primers 8F: 5′-ACTCCTACGGGAGGCAGCA-3′ and 149R: 5′-GGACTACHVGGGTWTCTAAT-3′, the contents of the colon was denatured at 98 °C for 30 s, annealed at 54 °C for 5 s, and extended at 54 °C for 15 s; this was then repeated 25–30 times, followed by extension at 72 °C for 2 min and the amplification of the highly variable regions V3-V4 of bacterial 16S rRNA. PCR products were sequenced on the Illumina MiSeq platform, and data were processed using the QIIME2 pipeline for sequence denoising or operational taxonomic unit (OTU) clustering. The organization and composition of the microbial community were then evaluated using α and β diversity analyses.

### 4.10. SCFAs Analysis

The quantities of SCFAs in the contents of the mouse colon were ascertained using gas chromatography–mass spectrometry (GC-MS). An Agilent HP-INNOWAX capillary column (30 m × 0.25 mm ID × 0.25 μm film thickness) combined with a 1 μL injection at a 10:1 split ratio was used for the analyses. The GC program set the inlet at 250 °C, the ion source at 300 °C, and the transfer line at 250 °C. A 90 °C temperature ramp was used to begin the temperature program. The heating protocol began at 90 °C and increased by 10 °C each minute to 120 °C, then gradually increased to 150 °C and then quickly to 250 °C. The helium carrier gas flow rate was kept constant throughout the chromatographic separation process at 1.0 mL/min. Mass spectrometric detection was conducted using a Thermo ISQ LT system (Thermo Fisher Scientific, Waltham, MA, USA) operated in electron impact ionization mode under specific conditions: 70 eV ionization energy with selective ion monitoring acquisition. Pure standards of acetic, butyric, propionic, isobutyric, isovaleric, and valeric acids (Sigma-Aldrich, St. Louis, MO, USA) were used for the identification and quantification of SCFAs.

### 4.11. Evaluating the Potentiating Effects of Butyrate on Ferroptosis in LLC Cells

#### 4.11.1. Cell Grouping and Treatment

LLC cells were plated in 6-well plates at 1 × 10^5^ cells per well and divided into six groups: Control, Rg1, Rh4, butyrate, butyrate + Rg1, and butyrate + Rh4. The control group was maintained in complete culture medium. The Rg1 and Rh4 groups were incubated with 100 μg/mL ginsenoside Rg1 and ginsenoside Rh4 for 24 h. The butyrate group was treated with butyrate (1.0 μM) for 24 h. The butyrate + Rg1 and butyrate + Rh4 groups were pretreated with butyrate (1.0 μM) for 24 h, followed by the addition of 100 μg/mL of ginsenoside Rg1 or ginsenoside Rh4, respectively, for an additional 24 h.

#### 4.11.2. RT-qPCR

The expression levels of selected genes were analyzed using RT-qPCR, following the method described in Section 4.4. The following primers were used for the amplification of target genes: *ATF3* (forward: 5′-CCTCGTCCCGTAGACAAAATG-3′, reverse: 5′-TTCTTGTTTCGACACTTGGCA-3′). The relative expression levels of these genes were quantified using the 2^−ΔΔCt^ method, with GAPDH serving as the internal control.

#### 4.11.3. Western Blotting

According to the Western blotting method in 2.7, the primary antibodies were *ATF3* (ImmunoWay Biotech, Plano, TX, USA), *SLC7A11* (Sangon Biotech, Shanghai, China), *GPX4* (Sangon Biotech, Shanghai, China), and β-actin (Proteintech, Wuhan, China). Protein expression levels were normalized to β-actin as a loading control.

### 4.12. Molecular Docking

The comprehensive chemical information of ginsenoside Rh4 was provided by the PubChem database (https://pubchem.ncbi.nlm.nih.gov/). The target protein *KEAP1* (PDB ID: 6TYM) was downloaded from the PDB database (http://www.wwpdb.org/). Prior to molecular docking, the protein was preprocessed using Schrodinger’s “Protein Preprocessing Wizard” tool, which added hydrogen atoms and missing side chains and ring structures while removing water molecules and ligands bound via hydrogen bonds. The grid was set to a 62 × 62 × 62 square grid. Finally, the treated proteins were fed into the optimized ligand ginsenoside Rh4 and XP was selected for docking to predict the docking binding results.

### 4.13. Statistical Analysis

GraphPad Prism 8.0.1, IBM SPSS Statistics 20.0, and OriginPro 2022 (OriginLab Corporation, Northampton, MA, USA) were used for all statistical analyses. Experimental results were presented as means ± standard deviation. Fisher’s LSD post hoc comparisons were used in one-way ANOVA to assess parametric datasets that confirmed normality. The thresholds for statistical significance were set at *p*-values < 0.05.

## 5. Conclusions

This study demonstrates that ginsenoside Rh4 exerts anti-tumor effects by inducing ferroptosis through modulating the *KEAP1*/*NRF2*/*HO-1* pathway, downregulating the *SLC7A11*/*GPX4* axis, and disrupting iron homeostasis. Simultaneously, ginsenoside Rh4 remodels the gut microbiota, increasing the abundance of butyrate-producing bacteria and elevating butyrate levels, which in turn enhances Rh4-induced ferroptosis through *ATF3*-mediated inhibition of *GPX4*. Collectively, these findings reveal a novel anti-tumor mechanism of ginsenoside Rh4 involving gut microbiota remodeling and the butyrate-mediated augmentation of ferroptosis.

## Figures and Tables

**Table 1:** The levels of short-chain fatty acids in feces samples.

Group	Acetic Acid (μg/g)	Propionic Acid (μg/g)	Isobutyric Acid (μg/g)	Butyric Acid (μg/g)	Isovaleric Acid (μg/g)	Valeric Acid (μg/g)	Caproic Acid (μg/g)
Control	917.2 ± 34.3	252.0 ± 43.4	17.4 ± 2.7	195.6 ± 24.1	14.6 ± 1.4	15.7 ± 1.4	1.3 ± 0.27
Model	588.9 ± 51.5 #	115.8 ± 15.7 ##	9.5 ± 1.5 ##	125.1 ± 15.6 ##	6.7 ± 1.1 #	9.5 ± 2.0 #	0.5 ± 0.10 ##
Rg1	638.6 ± 74.5	128.7 ± 6.5	10.2 ± 0.8	132.3 ± 28.5	7.5 ± 1.9	12.0 ± 0.9	0.7 ± 0.07
Rh4	796.5 ± 66.4	168.4 ± 19.6	14.8 ± 1.4 *	213.3 ± 21.6 **	9.3 ± 1.2	12.9 ± 3.1	0.8 ± 0.02

All data are expressed as mean ± SD (*n* = 4). Compared with the Control group, # *p* < 0.05, ## *p* < 0.01. Compared with the Model group, * *p* < 0.05, ** *p* < 0.01.

## Data Availability

The data presented in this study are available on request from the corresponding authors.

## Supplementary Materials

The following supporting information can be downloaded at: https://www.mdpi.com/article/10.3390/ijms27062703/s1.

## Abbreviations

The following abbreviations are used in this manuscript:

*ATF3*	Activating transcription factor 3
BCA	Bicinchoninic acid assay
CAT	Catalase
CCK-8	Cell counting kit-8
*FTH1*	Ferritin heavy chain 1
GC-MS	Gas chromatography–mass spectrometry
*GPX4*	Glutathione peroxidase 4
*GSH*	Glutathione
*HO-1*	Heme oxygenase-1
HPLC	High performance liquid chromatography
H&E	Hematoxylin–eosin
*KEAP1*	Kelch-like ECH-associated protein 1
KEGG	Kyoto encyclopedia of genes and genomes
LC-MS	Liquid chromatography–mass spectrometry
LefSe	Linear discriminant analysis effect size
LLC	Lewis lung carcinoma
*LPO*	Lipid peroxides
*MDA*	Malondialdehyde
NMR	Nuclear magnetic resonance spectroscopy
*NRF2*	Nuclear respiratory factor 2
NSCLC	Non-small cell lung cancer
OTU	Operational taxonomic unit
PBS	Phosphate-buffered saline
PCA	Principal component analysis
PCoA	Principal coordinates analysis
PVDF	Polyvinylidene fluoride
ROS	Reactive oxygen species
RT-qPCR	Real-time quantitative PCR
SCFAs	Short-chain fatty acids
*SLC40A1*	Solute carrier family 40 member 1
*SLC7A11*	Solute carrier family 7 member 11
SOD	Superoxide dismutase
*TFRC*	Transferrin receptor
