# Supplementary material for: Ginsenoside Rh4 Triggers Ferroptosis in Lung Cancer: Targeting KEAP1/NRF2/HO-1 and Remodeling Gut Microbiota for Butyrate-Mediated ATF3 Activation
Source: Int J Mol Sci. 2026 Mar 16;27(6):2703. doi: 10.3390/ijms27062703 (PMC13027123; doi:10.3390/ijms27062703)
Supplement: Supplementary file 1 [file ijms-27-02703-s001.zip › ijms-4108527- Supplementary file.pdf]

Supplementary Materials

Ginsenoside Rh4 Triggers Ferroptosis in Lung Cancer: Targeting KEAP1/NRF2/HO-1 and Remodeling Gut Microbiota for Butyrate-Mediated ATF3 Activation

Qihan Zhu[a,b], Wenxuan Xu[a], Ge Yang[b], Yansong Gao[b], Yujuan Zhao[b], Zijian Zhao[b], You Kang[b], Shengyu Li[b,*], Lei Zhao[a,*]

Institutions:

[a] School of Pharmaceutical Sciences, Changchun University of Chinese Medicine, Changchun 130117, P. R. China.

[b] Institute of Agro-food Technology, Jilin Academy of Agricultural Sciences (Northeast Agricultural Research Center of China), Changchun 130033, P. R. China.

*Corresponding authors:

Shengyu Li

Institute of Agro-food Technology, Jilin Academy of Agricultural Sciences, No. 1363 Sheng-Tai Street, Changchun, Jilin Province, 130033, P. R. China.

Tel.: +86 431 87063289

Fax: +86 431 87063075

E-mail: lisy720@126.com

Lei Zhao

School of Pharmaceutical Sciences, Changchun University of Chinese Medicine, Changchun 130117, P. R. China.

Tel.: +86 431 86045066

Fax: +86 431 86045066

E-mail: zhaolei@ccucm.edu.cn

Supplementary Figure Legends

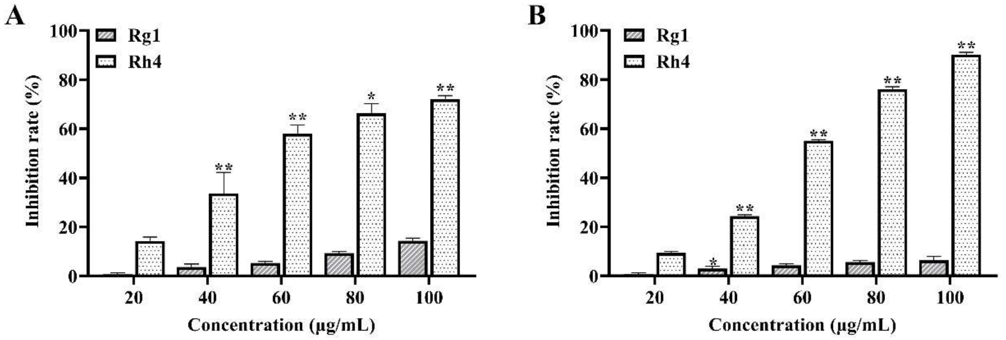

Figure supplementary S1 The effects of ginsenosides Rg1 and Rh4 on lung cancer cells (A) The proliferation inhibition rates of LLC treated with ginsenosides Rg1 or Rh4 for 24 hours; (B) The proliferation inhibition rates of A549 treated with ginsenosides Rg1 or Rh4 for 24 hours.

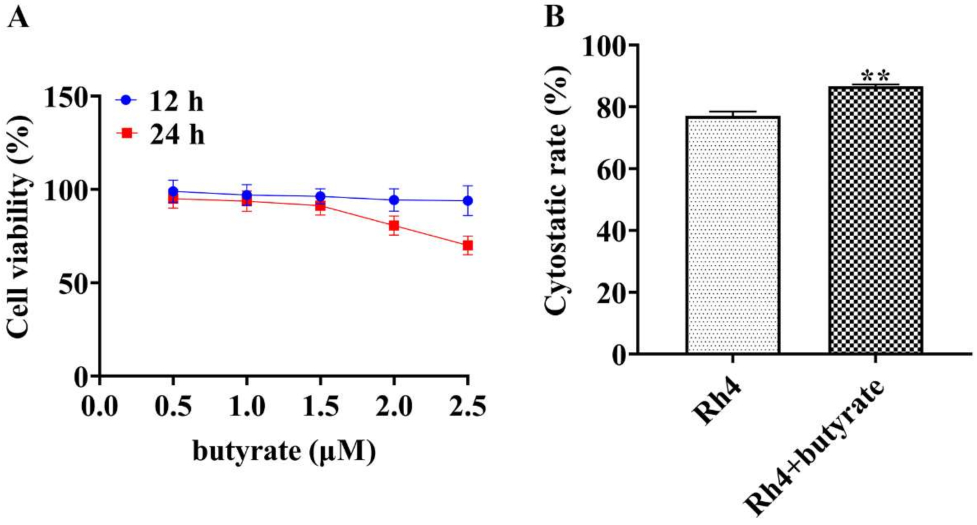

Figure supplementary S2 Butyrate synergistically enhances the inhibitory effect of ginsenoside Rh4 on LLC cells (A) Effects of different concentrations of butyrate on LLC cells. (B) The Effect of butyrate addition on the inhibition of LLC cells by ginsenoside Rh4.

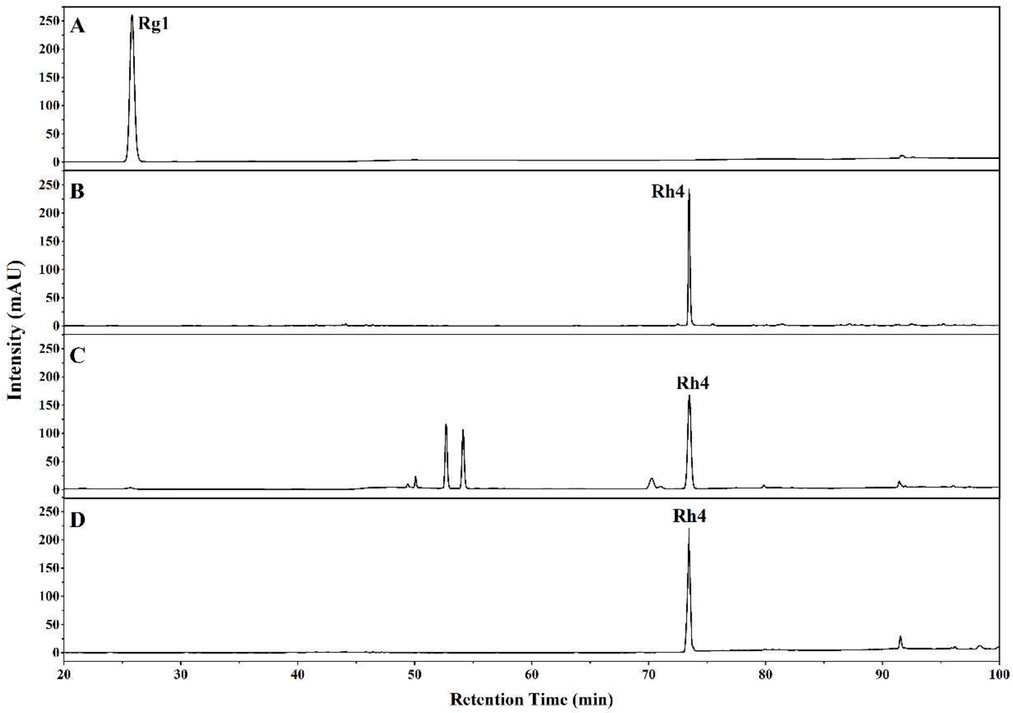

Figure supplementary S3 HPLC chromatogram of ginsenoside Rg1 fermented by *Lactobacillus plantarum* TRG22 to produce ginsenoside Rh4. (A) HPLC chromatogram of the ginsenoside Rg1 reference standard; (B) HPLC chromatogram of ginsenoside Rh4 standard; (C) HPLC chromatogram of ginsenoside Rg1 after fermentation; (D) HPLC chromatogram of ginsenoside Rh4 (1 mg/mL) derived from the fermentation product following macroporous resin separation and lyophilizationq.

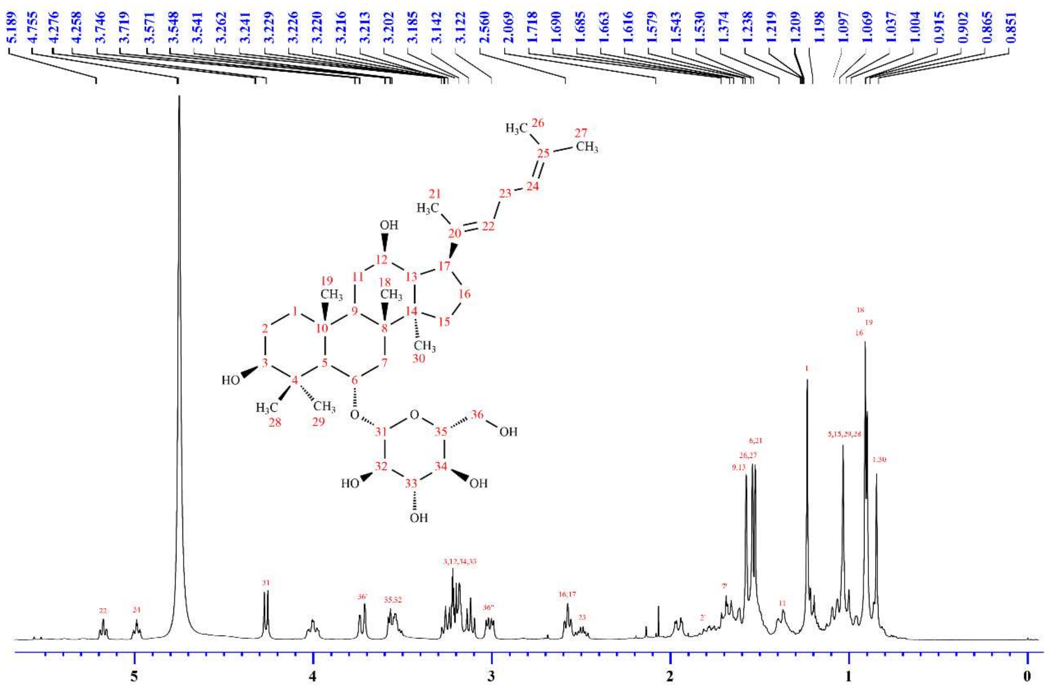

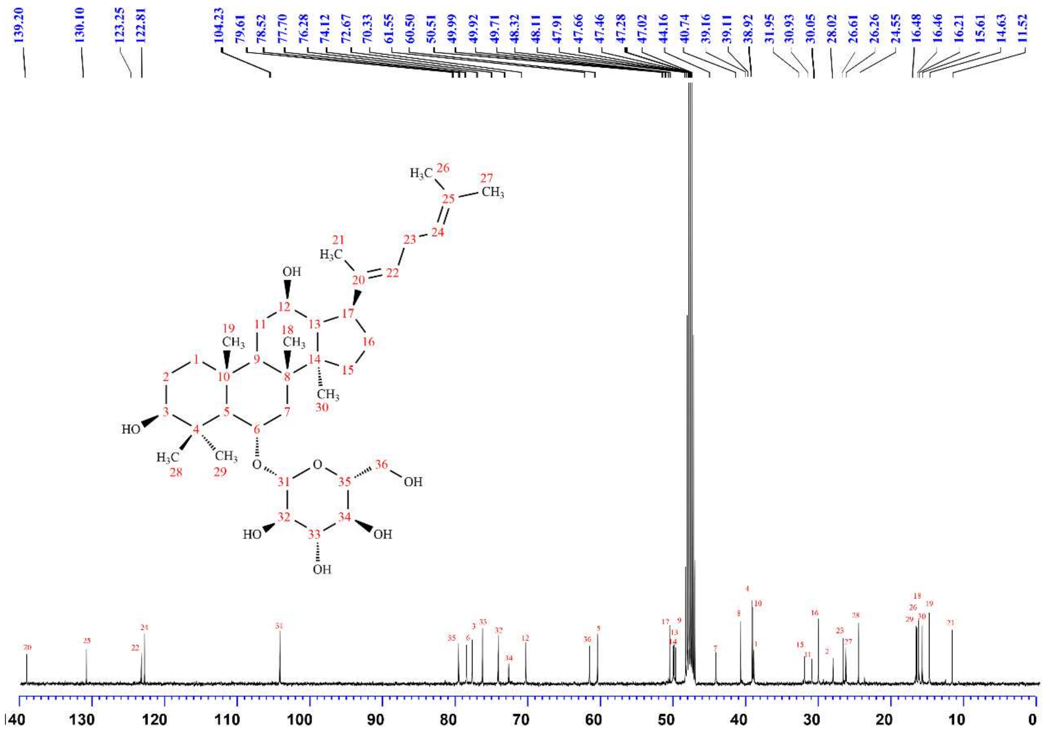

Figure supplementary S4 ^{1}H-NMR and ^{13}C-NMR spectra of ginsenoside Rh4
